# Readiness of healthcare providers for eHealth: the case from primary healthcare centers in Lebanon

**DOI:** 10.1186/s12913-016-1896-2

**Published:** 2016-11-10

**Authors:** Shadi Saleh, Rawya Khodor, Mohamad Alameddine, Maysa Baroud

**Affiliations:** Department of Health Management and Policy, American University of Beirut, Riad El Solh, PO Box 11–0236, Beirut, 1107 2020 Lebanon

**Keywords:** Healthcare providers, Primary healthcare, eHealth, eHealth tools, Readiness assessment

## Abstract

**Background:**

eHealth can positively impact the efficiency and quality of healthcare services. Its potential benefits extend to the patient, healthcare provider, and organization. Primary healthcare (PHC) settings may particularly benefit from eHealth. In these settings, healthcare provider readiness is key to successful eHealth implementation. Accordingly, it is necessary to explore the potential readiness of providers to use eHealth tools. Therefore, the purpose of this study was to assess the readiness of healthcare providers working in PHC centers in Lebanon to use eHealth tools.

**Methods:**

A self-administered questionnaire was used to assess participants’ socio-demographics, computer use, literacy, and access, and participants’ readiness for eHealth implementation (appropriateness, management support, change efficacy, personal beneficence). The study included primary healthcare providers (physicians, nurses, other providers) working in 22 PHC centers distributed across Lebanon. Descriptive and bivariate analyses (ANOVA, independent *t*-test, Kruskal Wallis, Tamhane’s T2) were used to compare participant characteristics to the level of readiness for the implementation of eHealth.

**Results:**

Of the 541 questionnaires, 213 were completed (response rate: 39.4 %). The majority of participants were physicians (46.9 %), and nurses (26.8 %). Most physicians (54.0 %), nurses (61.4 %), and other providers (50.9 %) felt comfortable using computers, and had access to computers at their PHC center (physicians: 77.0 %, nurses: 87.7 %, others: 92.5 %). Frequency of computer use varied. The study found a significant difference for personal beneficence, management support, and change efficacy among different healthcare providers, and relative to participants’ level of comfort using computers. There was a significant difference by level of comfort using computers and appropriateness. A significant difference was also found between those with access to computers in relation to personal beneficence and change efficacy; and between frequency of computer use and change efficacy.

**Conclusion:**

The implementation of eHealth cannot be achieved without the readiness of healthcare providers. This study demonstrates that the majority of healthcare providers at PHC centers across Lebanon are ready for eHealth implementation. The findings of this study can be considered by decision makers to enhance and scale-up the use of eHealth in PHC centers nationally. Efforts should be directed towards capacity building for healthcare providers.

## Background

The World Health Organization (WHO) defines eHealth as “the use of information and communication technologies (ICT) for health” [1]. A delivery platform that has been argued to particularly benefit from eHealth is primary healthcare (PHC). PHC aims to provide equitable, essential healthcare for all through patient centeredness, evidence-based medicine, and prevention and health promotion; its advantages include safer, more effective and efficient care, and ultimately, universal health coverage [2, 3]. Implementation of ICTs in PHC settings have resulted in time savings, improved access to results and data, decreased staff working time, greater accuracy, and increased ease and speed when retrieving patient data [4–7]. In other settings, eHealth can positively impact the efficiency and quality of healthcare services. At the organizational level, it may enhance efficiency by reducing duplication of diagnostic and therapeutic interventions, and by improving the automation of processes [8–10]. eHealth enhances flexibility, allowing integrated care centers [11]. For healthcare providers, it results in easier access and use of evidence-based guidelines with the potential to improve adherence and subsequently decrease medication errors [12]. Moreover, eHealth facilitates professional development, ultimately supporting recruitment and retention of healthcare providers in rural areas [11, 13]. For patients, eHealth provides them with the opportunity to become active participants in their own care [8]. For example, it can be used to assist older adults in facing their daily life difficulties [14].

The perceived benefits of eHealth carry a great promise for healthcare. However, in order to reap the benefits of this technology, it is important to address factors related to organizational readiness and provider readiness for eHealth. eHealth readiness is defined as the “preparedness of healthcare institutions or communities for the anticipated change brought by programs related to ICT” [15]. As with every organizational change, a major facilitating factor for the proper implementation and success of eHealth is in its adoption at the level of the organization [16]. Organizations may fail to successfully implement eHealth as a result of lack of readiness [17, 18]. A study to assess the readiness of healthcare providers working at a rehabilitation center in Canada emphasized the importance of both organizational and healthcare provider readiness for successful implementation [19].

At the organizational level, factors such as the simplicity of the system, early involvement of users, use of relevant terminology, proper education and training, and coordination with concerned stakeholders are all important factors that impact readiness for eHealth implementation [20–23]. Organizational barriers to implementation may also include workload, access to computers, and lack of support [24]. At the healthcare provider level, readiness and acceptance are key to successful eHealth implementation. A study from Afghanistan, conducted in hospitals in two different regions, found that readiness differed significantly between the hospitals included in the study due to the extent of usage of the technologies by providers [25]. A number of studies stressed the importance of assessing healthcare provider readiness for the implementation of ICTs in healthcare institutions [26, 27]. In Pakistan, stakeholders agreed that assessing readiness was essential to successful implementation [26].

Studies have revealed a number of factors that can potentially influence the readiness and willingness of healthcare providers to use eHealth, these include their perceptions concerning usefulness of the technology, resource and information availability, security of the system, their current level of ICT use, and whether they receive the necessary training to use the technology [14, 28–30]. Readiness to accept ICTs is also influenced by user computer literacy, which directly influences whether users view these technologies positively [31–33].

One comprehensive tool to evaluate healthcare providers’ readiness for implementation of the eHealth system at the PHC level is the “Readiness for Organizational Change” framework, which can be adapted to any organizational change and setting [34]. The tool covers many of the barriers addressed in the literature through four factors, and is useful for identifying individual level factors associated with change [34]. It assesses the providers’ perceptions of the “appropriateness” of the eHealth applications, whether they believe that there is “management support” for eHealth, whether they believe they have the skills or ability, termed “change efficacy”, to implement these applications, and whether they perceive the implementation of eHealth as “personally beneficial” to them [34].

The purpose of this study was to assess the readiness of healthcare providers working in PHC centers across Lebanon to use eHealth tools and applications.

### Context of Lebanon

Lebanon as a developing, upper-middle income country had a gross domestic product (GDP) at market prices of $45.73 billion in 2014 [35]. Total health expenditure as percentage of GDP in 2012 was estimated at 7.5 % [36]; this is due to the high dependence on the private sector for healthcare services [37, 38]. The public share of total health expenditure was estimated at 46.30 % in 2012, while the private share of total health expenditure was estimated to be 53.70 % in 2012, entailing a heavy burden on household income with the highest burden falling on those households in the lowest income category [36, 37]. Reflecting on this current challenge encountered by the Lebanese healthcare system, the Ministry of Public Health (MoPH) has expanded its focus to strengthening the primary care sector [38]. As for the number of physicians per hundred thousand, it was estimated to be 32.0 per 10,000 individuals [36]. In 2013, there were 3.1 hospitals/10,000 individuals [36].

As compared to other countries in the Eastern Mediterranean Region (average life expectancy of 73.4 years), Lebanon (2013) has a high life expectancy age of 80 years [36]. While in in the region, the average infant mortality rate stands at 42.5 deaths per 1000 individuals, the infant mortality rate in Lebanon currently (2013) stands at 7.8 deaths per 1000 individuals [36]. This low rate of infant mortality in Lebanon echoes the efforts made towards strengthening the basic and primary healthcare services in the country [39]. The total adult literacy rate between 2007 and 2012 was 90.0 %, while the total youth (15 to 24 years) literacy rate in 2007 was 98.7 % [36, 40].

There are over 800 PHC centers dispersed across all six governorates of Lebanon, two-thirds of which are owned and managed by non-governmental organizations [41]. Lebanon’s official primary healthcare network is comprised of 213 of these centers that have a contractual agreement with the MoPH based on pre-met community care delivery standards, which has enhanced the technical and organizational skills of these PHC centers in particular [42]. Services provided at these centers focus on promotion and prevention, and include general medicine services, such as pediatrics, cardiology, reproductive and oral health, among others. The centers also provide immunization services and drugs, as well as health and nutrition education to patients. PHC services are the predominant services provided at the community level in Lebanon [43].

The use of ICT applications in these centers is still lagging at the national level and for PHC centers. However, the MoPH is currently designing and introducing several eHealth tools in the 213 centers that comprise its network. To this purpose, the MoPH has been considering the provision of training on eHealth technologies for all healthcare providers, at the level of PHC. Training for administrators has already been implemented. These eHealth technologies include electronic medical records, basic decision support systems and disease reporting. This is possible because PHC centers under the MoPH network have the computers, hardware, and the necessary technical support for their use. However, the implementation of eHealth at the level of PHC centers is contingent on healthcare provider readiness.

## Methods

### Study design

This study assessed the readiness of healthcare providers working at PHC centers across Lebanon to use eHealth applications by using a cross-sectional study, employing a self-administered questionnaire with closed-ended questions. Ethical approval to conduct the study was obtained from the American University of Beirut Institutional Review Board (Protocol number: FHS.SS.13).

### Study tool

The study instrument comprised a quantitative questionnaire developed by Holt et al. to assess the readiness for change of employees within organizations [34]. The questionnaire can be modified and used in multiple settings [44]. It covers factors such as appropriateness of the change, support from leaders for the change, change efficacy, or the extent to which employees believe they can adopt the change, and whether the employees believe the change is beneficial to them personally [34]. The questionnaire touches on a number of the factors previously identified from the literature that impact the readiness of primary healthcare providers to use eHealth applications. A definition of eHealth was provided both in the questionnaire and the consent form as “the use of modern information and communication technologies to meet needs of citizens, patients, healthcare professionals, healthcare providers, as well as policy makers” [45].

The self-administered questionnaire consisted of three sections. The first section included questions related to the participants’ socio-demographics (gender, age, occupation, employment status, level of education, number of years of practice, and length of time working at the specific PHC center). The second section (4 questions) assessed the participants’ computer use, computer literacy, and whether they had access to computers at work. The final section of the questionnaire (26 questions) was adapted from the “Readiness for Organizational Change” scale for individuals, a reliable, validated scale, which assesses healthcare providers’ readiness for implementation of the eHealth system [34]. Holt et al. developed the scale inductively, and tested content adequacy, factor analysis (four factors emerged, variance: 62.7 %), convergent validity (readiness factors were found to be correlated with each other), and internal consistency (Cronbach’s alphas: .94 for appropriateness, .87 for management support, .82 for change efficacy, and .66 for personal valence) to end up with the 26 items included in the final scale [34]. Questions were ranked on a 6-point Likert scale, ranging from “1” = extremely disagree to “6” = extremely agree, and tackled four factors, which influence a participants’ readiness for change, in this case readiness for implementation of eHealth applications. The first factor, termed “appropriateness”, assesses appropriateness of the eHealth applications as perceived by the participant, in terms of whether they believe that the applications are appropriate within their context (10 questions). The second factor, termed “management support”, assesses whether participants believe management and leadership support, and are committed to the implementation of these eHealth applications (6 questions). The third factor, termed “change efficacy”, assesses whether participants believe they have the skills or the ability to implement the eHealth applications (6 questions). The fourth factor, “personally beneficial”, assesses whether the participants believe they will benefit from the eHealth applications (4 questions) [34].

The questionnaire was translated to modern Arabic. It was piloted for healthcare providers at a PHC center in Lebanon, and was adjusted accordingly. It was reviewed and validated by an expert panel, after translation to modern Arabic. Back-translation was conducted to ensure accurate translation of the tool.

### Participant selection and data collection

The MoPH provided a list of all operating PHC centers in the six governorates of Lebanon. At the time of the study, there were 213 centers operating within the MoPH network. The study population was estimated to be 3,200 healthcare providers. The sample size was calculated using a confidence level of 95 %, a response distribution of 50 %, and a margin of error of 5 %. It was therefore determined that the sample size should be 344 participants. Accounting for a desired response rate of at least 40 %, the final number of participants to be included in the study in order to ensure statistical significance as calculated, was estimated at 550. Proportional random sampling was used to select PHC centers in each governorate in order to ensure geographic representation. As such, 22 PHC centers were included in the study. The distribution of centers was as follows: 2 PHC centers from the Beirut governorate, 5 PHC centers from the Mount Lebanon governorate, 5 PHC centers from the North Lebanon governorate, 4 PHC centers from the Beqaa governorate, 3 PHC centers from the South Lebanon governorate, and 3 PHC centers from the Nabatiyeh governorate. All primary healthcare providers practicing at the selected centers were eligible for inclusion in the study (*n* = 541), irrespective of their occupation, employment status, and length of time employed at the specific PHC. These included physicians, nurses, technicians, pharmacists, and nutritionists.

Directors of the PHC centers were notified about the study via a memorandum, drafted and signed by the MoPH. Once their approval was obtained, research team members personally distributed questionnaire packages, including the written informed consent forms, provided in Arabic, and the questionnaires, also in Arabic, to the PHC. All centers received one telephone reminder two weeks after questionnaire distribution. One month after distribution, members of the research team personally picked up the questionnaires. Only questionnaires for which consent was obtained from participants were included in the analysis.

### Statistical analysis

The reliability of each factor in the questionnaire was assessed using Cronbach’s Alpha. Furthermore, descriptive and bivariate analyses (ANOVA, independent *t*-test, Kruskal Wallis, Tamhane’s T2) were used to compare participant characteristics to the level of readiness of healthcare providers from all governorates in Lebanon for implementation of the eHealth system. We considered the association between computer use, access to computers and computer literacy and readiness for implementation of the eHealth system, by assessing their relationship to the four factors previously described. Scores for each factor were calculated by determining the mean of the factor. This was computed by summing the scores of the respective item, and then dividing by the number of items. Missing data was accounted for using multiple imputation. The resulting score ranged from 1 to 6, with 6 referring to ‘extremely agree’. The authors interpreted a mean score above 4 for a factor to indicate that the group of respondents is ready for eHealth implementation. All factors were given equal weights in downstream statistical analyses. SPSS 21 was used for data analysis. A 95 % confidence interval and *P*-value ≤ 0.05 was used for statistical analysis.

## Results

Of the 541 questionnaires that were distributed, 213 were completed, rendering a response rate of 39.4 %. The majority of participants were physicians (46.9 %), followed by nurses (26.8 %) and other providers (24.9 %) (Table 1). This is in line with the actual distribution of healthcare providers in PHC centers in Lebanon, which usually employ more physicians than nurses and other health providers. With respect to employment status, 72.2 % of physicians work at the PHC center on a part-time basis compared to 7.0 % of nurses. As for other healthcare providers, the majority were full-time employees (75.5 %).

**Table 1:** Participants’ demographics and computer use characteristics

Characteristics	Nurses (*n* = 57)	Physicians (*n* = 100)	Others (*n* = 53)	Total
Gender				
Male	4 (7.0 %)	65 (65.0 %)	16 (30.2 %)	85
Female	47 (82.5 %)	27 (27.0 %)	37 (69.8 %)	111
Not specified	6 (10.5 %)	8 (8.0 %)	-	14
Age groups				
25–35	25 (43.9 %)	15 (15.0 %)	29 (54.7 %)	69
36–45	15 (26.3 %)	30 (30.0 %)	12 (22.7 %)	57
46–55	11 (19.3 %)	30 (30.0 %)	10 (18.9 %)	51
> 56	4 (7.0 %)	22 (22.0 %)	2 (3.8 %)	28
Not specified	2 (3.5 % )	3 (3.0 %)	-	5
Employment status				
Full time	51 (89.5 %)	11 (11.0 %)	40 (75.5 %)	102
Part time	4 (7.0 %)	72 (72.2 %)	8 (15.1 %)	84
Temporary/Casual	1 (1.8 %)	16 (16.0 %)	4 (7.5 %)	21
Not specified	1 (1.8 %)	1 (1.0 %)	1 (1.9 %)	3
Total years of practice				
< 1	2 (3.5 %)	2 (2.0 %)	4 (7.5 %)	8
1–5	5 (8.8 %)	17 (17.0 %)	18 (34 %)	40
6–10	15 (26.3 %)	15 (15.0 %)	7 (13.2 %)	37
11–15	10 (17.5 %)	17 (17.0 %)	10 (18.9 %)	37
16–20	5 (8.8 %)	10 (10.0 %)	5 (9.4 %)	20
> 20	20 (35.1 %)	38 (38.0 %)	8 (15.1 %)	66
Not specified	-	1 (1.0 %)	1 (1.9 %)	2
Total years of practice at respective PHC center				
< 1	6 (10.5 %)	9 (9.0 %)	6 (11.3 %)	21
1–5	9 (15.8 %)	37 (37.0 %)	20 (37.7 %)	66
6–10	17 (29.8 %)	24 (24.0 %)	6 (11.3 %)	47
11–15	7 (12.3 %)	16 (16.0 %)	11 (20.8 %)	34
16–20	4 (7.0 %)	5 (5.0 %)	2 (3.8 %)	11
> 20	14 (24.6 %)	9 (9.0 %)	6 (11.3 %)	29
Not specified	-	-	2 (3.8 %)	2
Comfort using computers				
Very comfortable	14 (24.6 %)	38 (38.0 %)	22 (41.5 %)	74
Comfortable	35 (61.4 %)	54 (54.0 %)	27 (50.9 %)	116
Not comfortable	8 (14.0 %)	7 (7.0 %)	1 (1.9 %)	16
Not specified	-	1 (1.0 %)	3 (5.7 %)	4
Access to computers at respective PHC center				
Yes	50 (87.7 %)	77 (77.0 %)	49 (92.5 %)	176
No	6 (10.5 %)	13 (13.0 %)	2 (3.8 %)	21
Not specified	1 (1.8)	10 (10.0 %)	2 (3.8 %)	13
Frequency of computer use at respective PHC center				
Monthly	4 (7.0 %)	6 (6.0 %)	-	10
Weekly	4 (7.0 %)	24 (24.0 %)	6 (11.3 %)	34
Daily	41 (71.9 %)	23 (23.0 %)	36 (67.9 %)	100
Never	8 (14.0 %)	42 (42.0 %)	10 (18.9 %)	60
Not specified	-	5 (5.0 %)	1 (1.9 %)	6
Sharing computers with colleagues				
Yes	43 (75.4 %)	47 (47.0 %)	25 (47.2 %)	115
No	13 (22.8 %)	46 (46.0 %)	26 (49.1 %)	85
Not specified	1 (1.8 %)	7 (7.0 %)	2 (3.8 %)	10

The majority of physicians were either very comfortable (38.0 %) or comfortable (54.0 %) using computers. As for nurses, 24.6 % were very comfortable using computers, while 61.4 % were comfortable using computers. In terms of other healthcare providers 41.5 % felt very comfortable using computers while 50.9 % felt comfortable using computers. Most healthcare providers (77.0 % of physicians, 87.7 % of nurses, and 92.5 % of other providers) had access to computers at their respective PHC centers. As for their frequency of computer use, 42.0 % of physicians never used the PHC center computer, whereas 71.9 % of nurses and 67.9 % of other types of healthcare providers use the computer on a daily basis.

### Reliability tests

Cronbach’s alpha values for the first factor, “appropriateness”, and the fourth factor, “personally beneficial” were 0.483 and 0.634, respectively. Such low values indicate issues with internal consistency and reliability concerning related items. As for the second factor, “management support”, and the third factor, “change efficacy”, the alpha values were 0.89 and 0.822, respectively. Such values suggest a high level of internal consistency and reliability of related items.

### Participant’s readiness for eHealth

Based on the results of the questionnaire (Table 2), there was no significant difference for the means of the factors, “appropriateness”, “management support”, “change efficacy”, and “personally beneficial”, for the different age groups. Similarly, there was no significant difference in the mean for the latter factors in relation to participants’ gender, highest level of education, total years of practice, and total years of practice at their respective PHC center. Concerning sharing the computer with colleagues at the respective PHC centers, there was no significant difference in the means for any of the four factors.

**Table 2:** Participant characteristics versus readiness for implementation of the eHealth system

	Factors			
	Appropriateness	Management support	Change efficacy	Personally beneficial
Age^b^ Mean (Standard deviation)				
25–35	5.01 (±0.64)	5.16 (±0.91)	5.25 (±0.79)	5.23 (±0.77)
36–45	5.06 (±1.01)	5.03 (±0.75)	4.99 (±0.90)	5.06 (±0.93)
46–55	5.09 (±0.51)	5.28 (±0.74)	5.16 (±0.11)	5.24 (±0.91)
> 56	5.00 (±0.69)	5.30 (±0.64)	4.95 (±0.95)	5.18 (±0.90)
Gender^a^ Mean (Standard deviation)				
Male	5.03 (±0.95)	5.06 (±0.79)	5.11 (±0.85)	5.15 (±0.93)
Female	5.06 (±0.54)	5.28 (±0.79)	5.15 (±0.86)	5.21 (±0.81)
Occupation^b^ Mean (Standard deviation)				
Physician	5.03 (±0.87)	5.01 (±0.78)^c^	5.12 (±0.82)	5.26 (±0.80)
Nurse	4.97 (±0.57)	5.11 (±0.86)	4.85 (±0.90)^d^	4.80 (±0.96)^e, f^
Others	5.16 (±0.58)	5.47 (±0.70)	5.38 (±0.77)	5.44 (±0.74)
Employment status at the respective PHC center^b^ Mean (Standard deviation)				
Full-time	5.09 (±0.84)	5.32 (±0.79)^g^	5.09 (±0.88)	5.07 (±0.94)
Part-time	4.99 (±0.59)	5.05 (±0.76)	5.12 (±0.87)	5.29 (±0.79)
Temporary/Casual	4.88 (±0.73)	4.70 (±0.76)	5.08 (±0.72)	5.19 (±0.78)
Highest level of education^b^ Mean (Standard deviation)				
High school graduate	5.40 (±0.28)	5.40 (±0.93)	5.38 (±0.58)	5.45 (±0.81)
Technical school graduate	4.86 (±0.71)	5.08 (±0.90)	4.87 (±0.90)	4.76 (±0.96)
Haven’t graduated university	5.26 (±0.27)	5.46 (±0.56)	5.16 (±0.92)	5.45 (±0.60)
Undergraduate degree	5.05 (±0.52)	5.26 (±0.73)	5.12 (±0.90)	5.25 (±0.92)
Graduate degree	5.36 (±1.20)	5.44 (±0.59)	5.54 (±0.76)	5.35 (±0.82)
Doctoral degree	4.91 (±0.64)	4.88 (±0.78)	5.02 (±0.83)	5.21 (±0.81)
Other	5.15 (±0.56)	5.66 (±0.66)	4.58 (±1.03)	5.25 (±0.54)
Total years of practice^b^ Mean (Standard deviation)				
Less than one year	5.17 (±0.44)	4.90 (±0.46)	5.07 (±0.62)	5.25 (±0.43)
Between 1 and 5 years	5.14 (±1.13)	5.01 (±0.95)	5.23 (±0.83)	5.26 (±0.75)
Between 6 and 10 years	4.93 (±0.67)	5.20 (±0.91)	5.08 (±0.87)	5.25 (±0.75)
Between 11 and 15 years	5.03 (±0.61)	5.23 (±0.80)	5.16 (±0.78)	5.32 (±0.87)
Between 16 and 20 years	5.06 (±0.41)	5.34 (±0.64)	5.07 (±0.94)	4.93 (±1.00)
More than 20 years	5.00 (±0.63)	5.20 (±0.71)	5.00 (±0.92)	5.03 (±1.00)
Total years of practice at the respective PHC center^b^ Mean (Standard deviation)				
Less than 1 year	4.95 (±0.66)	4.76 (±0.84)	5.09 (±0.66)	5.21 (±0.65)
Between 1 and 5 years	5.13 (±0.91)	5.15 (±0.84)	5.21 (±0.80)	5.33 (±0.76)
Between 6 and 10 years	4.94 (±0.69)	5.17 (±0.83)	4.95 (±0.94)	5.01 (±0.91)
Between 11 and 15 years	5.20 (±0.51)	5.41 (±0.69)	5.40 (±0.70)	5.28 (±0.95)
Between 16 and 20 years	4.94 (±0.54)	4.77 (±0.79)	4.95 (±1.11)	5.18 (±0.92)
More than 20 years	5.03 (±0.74)	5.17 (±0.80)	5.10 (±0.86)	5.18 (±0.87)

^a^Independent *T*-test

^b^Anova One-way analysis of means, with Bonferroni multiple comparison test, and Kruskal-Wallis One-way analysis of variance, with

Tamhane T2 analysis of unequal variances were used for statistical analysis

^c, d, e^A significant difference was found with other healthcare providers

^f^A significant difference was found with physicians

^g^A significant difference was found with temporary/casual employees

#### Appropriateness

In terms of comfort using computers, those who were very comfortable and comfortable using computers believed that the eHealth system is appropriate for their PHC center, more than healthcare providers who were not comfortable using computers (*p* = 0.002 and *p* = 0.009, respectively).

#### Management support

Other healthcare providers recognize the commitment of the PHC management to the implementation of eHealth applications, more so than physicians (*p* = 0.007); however, there was no significant difference when compared to nurses. In general, when compared to temporary or casual employees, full-time healthcare providers have a greater belief that the PHC management and leadership are committed to implementation of the eHealth system (*p* = 0.014). There was no significant difference in relation to “management support” between physicians and nurses, and nurses and other healthcare providers. Furthermore, those who were very comfortable using computers, believe that there is management and leadership support for the eHealth system, more than those who were comfortable, and not comfortable using computers (*p* = 0.042 and *p* = 0.049, respectively).

#### Change efficacy

With respect to frequency of computer use, there was a significant difference between healthcare providers who use the computer at the PHC center daily, versus those who never use the computer at the PHC center, in relation to “change efficacy” (*p* = 0.01). There was a significant difference in the three levels of comfort using computers for the factors “change efficacy” and “personally beneficial”. Specifically, those that were very comfortable using computers believed that they have the skills and abilities for using the eHealth system once it is implemented, and that they will benefit from the eHealth system, as compared to those who were comfortable (*p* = 0.001 and *p* = 0.01, respectively), and not comfortable using computers (*p* = 0.000 and *p* = 0.008, respectively). Also, other healthcare providers believe that they have the skills and ability to implement the eHealth system, more so than nurses (*p* = 0.005). Moreover, other healthcare providers believe that they have the skills and ability to implement the eHealth system, more so than nurses (*p* = 0.005). In addition, those that have access to computers believe more than those that do not have access to computers that they have the skills and abilities to implement the eHealth system (*p* = 0.01).

#### Personally beneficial

There was a significant difference in the mean for the factor, “personally beneficial”, according to type of healthcare providers between physicians and nurses, and nurses and other healthcare providers (*p* = 0.005 and *p* = 0.001, respectively). Namely, physicians and other healthcare providers believe that they will personally benefit more from the eHealth system than nurses. Based on the results of the questionnaire (Table 3), there was a significant difference in belief concerning personal beneficence once the eHealth system is implemented among those that have access to computers at the PHC center, versus those that do not have access to computers (*p* = 0.022).

**Table 3:** Computer use versus readiness for implementation of the eHealth system

	Factor			
	Appropriateness	Management support	Change efficacy	Personally beneficial
	Mean (Standard deviation)			
Comfort using computers^b^				
Very comfortable	5.15 (±0.52)^c^	5.38 (±0.70)^e, f^	5.54 (±0.60)	5.44 (±0.67)
Comfortable	5.05 (±0.82)^d^	5.06 (±0.80)	5.05 (±0.82)	5.10 (±0.89)
Not comfortable	4.43 (±0.62)	4.74 (±1.02)	3.76 (±0.82)	4.38 (±1.01)
Access to computers at respective PHC center^a^				
Yes	5.08 (±0.74)	5.21 (±0.80)	5.20 (±0.79)^g^	5.24 (±0.84)^h^
No	4.88 (±0.57)	4.90 (±0.75)	4.48 (±0.99)	4.73 (±0.99)
Sharing computers with colleagues^a^				
Yes	5.04 (±0.85)	5.15 (±0.79)	5.12 (±0.79)	5.15 (±0.84)
No	5.08 (±0.54)	5.17 (±0.81)	5.14 (±0.92)	5.25 (±0.89)
Frequency of computer use at respective PHC center^b^				
Monthly	4.58 (±0.53)	4.97 (±0.90)	4.76 (±0.98)	5.05 (±0.69)
Weekly	5.21 (±1.20)	5.18 (±0.74)	5.12 (±0.77)	5.29 (±0.86)
Daily	5.10 (±0.57)	5.30 (±0.79)	5.31 (±0.75) ^i^	5.26 (±0.84)
Never	4.95 (±0.61)	4.90 (±0.77)	4.85 (±0.93)	5.01 (±0.94)

^a^Independent *T*-test

^b^Anova One-way analysis of means, with Bonferroni multiple comparison test, and Kruskal-Wallis One-way analysis of variance, with

Tamhane T2 analysis of unequal variances were used for statistical analysis

^c, d, e^A significant difference was found with those not comfortable using computers

^f^A significant difference was found with those comfortable using computers

^g, h^A significant difference was found between those with access to computers versus those who do not have access to computers

^i^A significant difference was found between those who use computers at the PHC center daily versus those who never use the computers

## Discussion

Many have argued that eHealth holds promise of enhanced efficiency and quality in healthcare [8, 9]. A facilitating factor for the adoption and successful implementation of eHealth is the acceptance and readiness of healthcare providers [17]. To our knowledge, no studies have been conducted in Lebanon and the Arab region to assess the readiness of primary healthcare providers for the implementation of eHealth applications. This paper attempts to address this knowledge gap. The results of this study demonstrate the extent of readiness of healthcare providers for eHealth implementation in PHC centers across Lebanon, as well as demographic, professional and organizational factors associated with such readiness.

The findings of this study revealed no relation between age of healthcare providers and their readiness for implementation of the eHealth system. This is in contrast to the findings of a study that found that older age groups have more positive attitudes regarding the usefulness of technologies than younger age groups [46]. Also, this study did not find a significant association between the gender of healthcare providers and their readiness for implementation of eHealth, in contrast to the findings of a study that identified that the degree of perceived usefulness among participants differed depending on gender [46]. This may be explained by our finding that the majority of healthcare providers expressed that they are comfortable or very comfortable using computers, irrespective of their age and gender. Literature has shown that computer knowledge and acceptance of, or readiness for, eHealth and other IT applications are positively correlated [28, 31, 47].

Study findings revealed no significant association between highest level of education and readiness to embrace eHealth among the participants. These findings are congruent with a study conducted among nurses [48]. Also, although approximately one third of the providers in this study practiced for more than 20 years, there was no significant association between total years of practice and readiness to use eHealth. Studies have shown that providers with greater than 20 years of practice were less likely to accept technologies [49, 50]. However, a study found that long-term care work experience did not have any significant effect on healthcare providers’ acceptance of ICT applications [21]. Moreover, physicians were more likely to believe that they will personally benefit from the eHealth system than nurses. Studies have shown that physicians are more likely to accept a technology when they believe it is beneficial and useful for their practice [51–53].

There was a significant difference between other healthcare providers and physicians concerning beliefs about management and leadership support for the implementation of the eHealth system. This may be related to the fact that the majority of other healthcare providers are employed on a full-time basis, while most physicians are employed on a part-time basis. Consistent with the latter, there was a significant difference between the employment status of healthcare providers, and their beliefs about “management support”. Spending less time in the workplace, as may be the case of a number of part-time healthcare providers, could result in communication problems with the organization, and may impact workplace relationships (Feldman, 1995); as such, part-time employees may not be aware of all management decisions.

The majority of healthcare providers included in the study have access to computers at their respective PHC centers. This finding is important, since among nurses, lack of access to computers acts as a major barrier to the adoption of ICTs in their workplace [24, 54]. Furthermore, our study found that healthcare providers (physicians, nurses and others) with access to computers believed that they would personally benefit from the implementation of an eHealth system, and that they have the skills for the implementation of such a system. A study conducted among healthcare providers in primary care practices found that providers that use computers often were more eager regarding technology, and that providers already using computers believed they had the skills for adapting to newly implemented technologies [28]. Another study showed that current ICT utilization among physicians acts as a facilitator for the acceptance and use of eHealth technologies [53]. Those healthcare providers who used the computer at the center on a daily basis, believed more that they had the necessary skills for the implementation of such a system, as compared to those who never used the computers at the center; this corroborates with findings from another study conducted in a primary healthcare center setting [28].

The findings from the study also demonstrated that healthcare providers who are very comfortable using computers agree that the eHealth system is appropriate for their center, and that they will personally benefit from the implementation of an eHealth system at their respective PHC centers. A number of studies have noted the relation between computer knowledge and the acceptance of, or readiness for eHealth and other ICT applications [28, 31, 47]. One study found that baseline levels of computer knowledge were related to eHealth readiness among healthcare providers [31], while other studies found that healthcare providers with past experiences using computers are positive about ICTs, and recognize their benefits [24, 28, 55]. As well, lack of computer skills and ICT knowledge may act as a barrier to the adoption of ICTs [56, 57].

### Policy and practice implications

The health care needs – and expectations – of populations have not been higher than in recent times. This is especially true in developing countries, which are witnessing a double burden of disease, communicable and non-communicable [58]. In light of this, health care systems in these countries must enhance their responsiveness and capacity to respond to such needs, which is a challenging undertaking given limited resources. eHealth can serve as an innovative and effective approach to help address this dilemma, especially since in recent years, there has been a significant enhancement of technological infrastructures in almost all countries, making eHealth a potentially affordable care delivery ‘supplement’ or ‘enhancer’ [59]. A natural setting for such innovative and effective tools is the PHC setting.

The PHC sector in Lebanon, similar to that in most developing countries, has endured decades (after the 1978 Alma Ata Declaration) of minimal attention by policy-makers. However, recent years have brought a renewed interest and belief in its potential, especially after the 2008 World Health Report, Primary Health Care: Now More than Ever [3]. In this region, the renewed interest has also been fueled by an acute, and lasting, refugee crisis that overburdened the already stretched health care system. It is in this context that the idea of this paper was developed to assess whether the primary potential users are ready/receptive.

Overall, the study found a very noteworthy readiness among providers, albeit a bit different across health care professions and by familiarity with technology. Such an enabling finding should encourage policy makers, especially the MoPH, to first enhance, and then scale up the implementation and use of eHealth in the care delivery model in PHC centers at a national level. As a practical recommendation, the authors believe that supporting health care providers at PHC clinics with computer-based up-to-date clinical guidelines for common illnesses would be feasible. This would ensure evidence-based treatment and a standardized approach for common clinical conditions, especially maternal and child health, as well as non-communicable diseases. In addition, as part of their mission, PHC centers have a role in community health. The use of eHealth, specifically mHealth (or mobile health), can serve a vital role in outreach activities such as health promotion, disease management, etc., among community dwellers [60]. Although this may not have the ability to affect all populations – illiteracy, no phones, etc. – it will play an important facilitating role in health outreach.

At the organizational level, PHC management should redirect their capacity building efforts towards the provision of trainings for the use of eHealth technologies. Furthermore, PHC centers may benefit from designating Champions whose responsibilities should include leading and guiding the implementation of eHealth tools. It is important to note that this cannot be achieved without a strong alignment between the MoPH’s vision for eHealth and the PHC management’s support for this vision.

## Conclusion

Effective eHealth implementation cannot be achieved if healthcare providers are not ready for such a change. This study found that the majority of healthcare providers at PHC centers across Lebanon are ready for eHealth implementation. The findings of this study can be considered by decision makers to enhance and scale-up the use of eHealth in PHC centers nationally. As such, efforts should be directed towards capacity building for healthcare providers and primary healthcare centers across the nation. Furthermore, the findings from this study can serve as a stepping-stone to a serious discussion among policy makers and practitioners on approaches to adopt eHealth into care delivery models. However, issues of equity of access and quality have to be taken into consideration in deciding on the way forward.

### Limitations

This study has a number of limitations. Firstly, the response rate was 39.37 % which may limit the generalizability of the study findings. Nonetheless, it has been found that surveys sent to physicians are characterized by a low response rate, due to lack of time, refusal to participate, perceived salience of the study, and confidentiality of the responses [61].

Another limitation is the non-inclusion of all the PHC centers in the study. However, PHC centers were evenly distributed across governorates ensuring fair representation. Finally, the low values of Cronbach’s alpha for the “appropriateness”, and “personally beneficial” factors can be considered one of the limitations of the study. However, since the MoPH is still in the process of designing and introducing eHealth to the PHC sector, it may be that most of the healthcare providers at the PHC centers are in the first stages of being introduced to eHealth implementation, or have not been exposed yet to such implementations. This may also be due to the fact that most of the healthcare providers are physicians who work at the centers on a part-time basis, which likely affects their intimate knowledge of the PHC centers, beliefs concerning the appropriateness of eHealth, as well as the perceived benefits of its applications.

## Abbreviations

GDP: Gross domestic product
ICT: Information and communication technologies
MoPH: Ministry of Public Health
PHC: Primary healthcare
WHO: World Health Organization
